# Genome-Wide Identification of the Dirigent Gene Family and Expression Pattern Analysis Under Drought and Salt Stresses of *Sorghum bicolor* (L.)

**DOI:** 10.3390/genes16080973

**Published:** 2025-08-19

**Authors:** Shipeng Liu, Tingrui Jing, Shuang Liang, Hairuo Wang, Xinyi Guo, Quan Ma, Junshen Wang, Kai Wang, Xiaolong He, Haibin Zhao, Wenting Jiang, Xiangqian Zhang

**Affiliations:** 1 College of Life Sciences, Yan’an University, Yan’an 716000, China; liushipeng2003@126.com (S.L.); asdfggfff@126.com (T.J.); liangs320@163.com (S.L.); 15129783562@163.com (H.W.); xwyxhh@126.com (X.G.); m15191134034@163.com (Q.M.); 15891783352@163.com (J.W.); hexiaolong@yau.edu.cn (X.H.); jiangwenting@yau.cdu.cn (W.J.); 2 Engineering Research Center of Microbial Resources Development and Green Recycling of Shaanxi Province, Yan’an University, Yan’an 716000, China; wangkai@yau.edu.cn (K.W.); zhbyanandaxue@163.com (H.Z.)

**Keywords:** *SbDIR* gene family, systematic evolution, tandem duplication, abiotic stresses

## Abstract

**Background:** The *Dirigent* (*DIR*) gene family is pivotal for lignin polymerization and stress adaptation in plants, yet its systematic characterization in *Sorghum bicolor* (*S. bicolor*), a critical bioenergy crop, remains underexplored. **Methods:** Leveraging the *S. bicolor* genome database, we conducted a genome-wide identification, phylogenetic classification, and expression profiling of the *DIR* gene family. Evolutionary dynamics, gene structure variations, promoter cis-regulatory elements, and spatiotemporal transcriptome patterns were analyzed using bioinformatics and experimental validation (RT-qPCR). **Results:** A total of 53 *SbDIR* genes were systematically identified, exhibiting uneven chromosomal distribution. Phylogenetic analysis clustered them into five clades (DIR-a, DIR-b/d, DIR-c, DIR-e, DIR-f), with subfamily-specific exon number variations suggesting functional divergence. Evolutionary studies revealed tandem duplication (TD) as the primary driver of family expansion, accompanied by strong purifying selection. Promoter analysis highlighted abundant hormone- and stress-responsive cis-elements. Tissue-specific RNA-seq data revealed root-enriched expression of *SbDIR2/4/18/39/44/53*, implicating their roles in root development. Notably, *SbDIR39* and *SbDIR53* were significantly upregulated (2.8- and 5-fold, respectively) under 150 mM NaCl stress, underscoring their stress-responsive functions. **Conclusions:** This study provides the first comprehensive atlas of the *DIR* gene family in *S. bicolor*, elucidating its evolutionary mechanisms and tissue-specific/stress-induced expression profiles. Key candidates (*SbDIR39/53*) were identified as promising targets for molecular breeding or CRISPR-based editing to enhance stress resilience in *S. bicolor*. These findings lay a foundation for translating genomic insights into agronomic improvements.

## 1. Introduction

Dirigent (DIR) proteins constitute a critical regulatory protein family in plant secondary metabolism, with their name derived from the Latin “dirigere,” meaning “to guide” or “to direct.” These proteins were first discovered in the lignin biosynthesis pathway of *Forsythia intermedia*, where they specifically facilitate the radical coupling reaction of coniferyl alcohol to form (+)- pinoresinol [1]. In the absence of DIR proteins, this reaction yields only racemic mixtures. This discovery highlighted the stereoselective regulatory role of DIR proteins in plant phenylpropanoid metabolism [2].

With the advent of multiple plant genome sequences, an increasing number of *DIR* genes have been identified, such as in *Thuja plicata* (*T. plicata*) [3], *Schisandra chinensis* (*S. chinensis*) [4], *Pisum sativum* (*P. sativum*) [5], *Linum usitatissimum* (*L. usitatissimum*) [6], Arabidopsis thaliana (*A. thaliana*) [7], Glycine max (*G. max*) [8], Oryza sativa (*O. sativa*) [9,10], *S. lycopersicum* (*Solanum lycopersicum*) [10], *Pigeonpea* (*Cajanus cajan* L.) [11], *M. bamboo* (*Moso bamboo*) [12], and *H. pedunculosum* (*Herpetospermum pedunculosum*) [13]. These findings have significantly expanded our understanding of the DIR protein family and established a solid foundation for further phylogenetic studies.

Research has demonstrated that DIR proteins not only participate in lignin biosynthesis but also play important roles in plant defense responses, abiotic stress adaptation, and developmental regulation. For example, in *A. thaliana* and *Zea mays* (*Z. mays*), *ESB1* and *ESBL* are involved in the development of the Casparian strip [14]. Additionally, when the moss *Physcomitrium patens* (*P. patens*) is infected by *Colletotrichum gloeosporioides* (*C. gloeosporioides*), defense genes such as *PAL*, *CHS*, and *DIR-like* are activated [15]. Knockout mutants of *OsDIR55* exhibit reduced NaCl tolerance, whereas overexpression lines show enhanced NaCl tolerance [16]. Moreover, *ZmDIR5* serves as a positive regulator of maize tolerance to waterlogging, salt, and drought stresses [17]. In *Glycine max* (*G. max*), the expression of *GmDIR22* responds to exogenous hormones such as GA3, SA, MeJA, and ABA [6,18]. Furthermore, transgenic *G. max* lines overexpressing *GmDIR22* exhibit enhanced resistance to *Phytophthora sojae* (*P. sojae*) by increasing lignan biosynthesis [18].

Previous phylogenetic analyses of DIR proteins in *Picea asperata* (*P. asperata*), *Saccharum officinarum* (*S. officinarum*), *O. sativa*, *A. thaliana*, *Gossypium hirsutum* (*G. hirsutum*), and *Hordeum vulgare* (*H. vulgare*) have classified these proteins into six subfamilies: DIR-a, DIR-b/d, DIR-c, DIR-e, DIR-f, and DIR-g [19,20]. Among these subfamilies, DIR-b/d, DIR-c, DIR-e, DIR-f, and DIR-g are categorized as DIR-like subfamilies, whereas DIR-a is classified under the DIR subfamily [19,20]. Notably, the DIR-c and DIR-f subfamilies are uniquely distributed among monocots. Some members of the DIR-c subfamily exhibit a distinctive domain architecture, characterized by an N-terminal DIR domain and a C-terminal jacalin (JAC) lectin domain [21,22]. This structure may confer unique biological functions to specific DIR-c subfamily members.

Comprehending the roles of *DIR* family genes in biological and physiological processes offers a viable avenue for analyzing and enhancing crop defenses against both biotic and abiotic stresses. *S. bicolor* exhibits strong stress tolerance, enabling it to grow under abiotic stresses such as drought, salinity, and poor soil conditions, thereby making it an ideal model for studying the mechanisms of plant stress resistance [23]. Although the *DIR* gene family has been extensively investigated in model plants such as *A. thaliana* and *O. sativa* [7,9,10], a systematic analysis of this gene family in *S. bicolor* remains lacking. Therefore, in this study, we conduct a comprehensive biological analysis of the *DIR* gene in *S. bicolor*, including phylogenetic relationships, conserved protein motifs, gene structures, cis-acting elements, tissue-specific expression, and expression levels in various tissues under different abiotic stresses. This research will provide a theoretical basis for future functional studies and enhance our understanding of the roles of *DIR* genes in the growth, development, and stress responses of *S. bicolor*.

## 2. Materials and Methods

### 2.1. Identification of DIR Gene Family Members in S. bicolor

The hidden Markov model (HMM) profile of the DIR domain (PF03018) was downloaded from the Pfam (https://pfam.xfam.org/, accessed on 10 April 2025) database [24] and used to search the *S. bicolor* genome database (Phytozome v13) (https://phytozome-next.jgi.doe.gov/, accessed on 10 April 2025) [25] using HMMER 3.0 with an E-value threshold of 1 × 10^−5^. Candidate gene sequences were submitted to the NCBI Conserved Domain Database (CDD) (https://www.ncbi.nlm.nih.gov/Structure/bwrpsb/bwrpsb.cgi, accessed on 10 April 2025) and the SMART (https://smart.embl.de/, accessed on 10 April 2025) database for domain validation. Sequences lacking complete DIR domains were removed, and the final set of *S. bicolor DIR* gene family members was determined.

### 2.2. Chromosomal Localization, Gene Structure, and Conserved Motif Analysis of SbDIR Genes

Based on *S. bicolor* genome annotation information, the distribution of *DIR* genes on chromosomes was mapped using TBtools v2.121 [26]. Exon–intron structures of *DIR* genes were analyzed with TBtools v2.121. Conserved motifs in *S. bicolor* DIR proteins were predicted using the MEME 5.4.1 online tool (https://meme-suite.org/meme/tools/meme, accessed on 20 April 2025) [27], with the number of motifs set to 10 and other parameters at default values.

### 2.3. Construction of the Phylogenetic Tree of DIR Proteins

Reported DIR protein sequences from *A. thaliana*, *Solanum lycopersicum* (*S. lycopersicum*), *Setaria italica* (*S. italica*), *O. sativa*, and *Z. mays* were aligned with *S. bicolor* DIR sequences using ClustalW 2. A neighbor-joining (NJ) phylogenetic tree was constructed with 1000 bootstrap replicates by MEGA 7.0 [28] and visualized using the iTOL v4 (https://itol.embl.de/, accessed on 20 April 2025) online tool.

### 2.4. Analysis of Cis-Regulatory Elements in SbDIR Gene Promoters

The 2000 bp upstream sequences of *S. bicolor DIR* gene start codons were extracted as promoter regions and submitted to the PlantCARE (https://bioinformatics.psb.ugent.be/webtools/plantcare/html/, accessed on 20 April 2025) [29] database to identify potential *cis*-acting elements and their distribution.

### 2.5. Tissue-Specific Expression and Stress Response Analysis of S. bicolor DIR Genes

Transcriptome data from 13 different developmental stages and tissues (including seedlings, leaves, roots, stems, inflorescences, and seeds) in the *S. bicolor* Genome and Mutant Database (SGMD, https://S. bicolor.genetics.ac.cn/SGMD, accessed on 25 April 2025) [30] were used to analyze the expression patterns of *DIR* genes across tissues. The data are shown in a heatmap with gene expression in different tissues with row-scaled transcriptome atlas (TPM values). Red and blue boxes indicate high and low expression levels of *SbCBL* genes.

### 2.6. Total RNA Extraction and RT-qPCR

*S. bicolor* was grown in a growth chamber at Yan’an University under 16 h of light and with a temperature of 25 °C and 70% humidity maintained. To determine the expression level of the *DIR* gene after NaCl and PEG treatments, *S. bicolor* seedlings at the three-leaf-one-heart stage were selected. The seedlings were then treated with 150 mM NaCl and 15% PEG, respectively. Root samples were collected from the seedlings seven days later. All experiments were performed in three biological replicates. Total RNA was isolated using the Plant Total RNA Kit from Beijing Zhuangmeng International BioGenetics Co., Ltd. (Beijing, China), and reverse transcription was performed using the HiScript IV All-in-One Ultra RT SuperMix for qPCR from Novozymes (Bagsværd, Denmark). The RT-qPCR amplification reaction system consisted of 5 μL of 2× SYBR, 3 μL of ddH_2_O, 1 μL of the cDNA template, and 0.5 μM of the forward and reverse primers, for a total volume of 10 μL. We analyzed the expression level of the *SbDIR* gene by the 2^−ΔΔCT^ method in response to different stress treatments, using *SbACTIN* as an internal reference gene.

## 3. Results

### 3.1. Identification and Chromosomal Localization of the DIR Gene Family in S. bicolor

**Figure 1:**
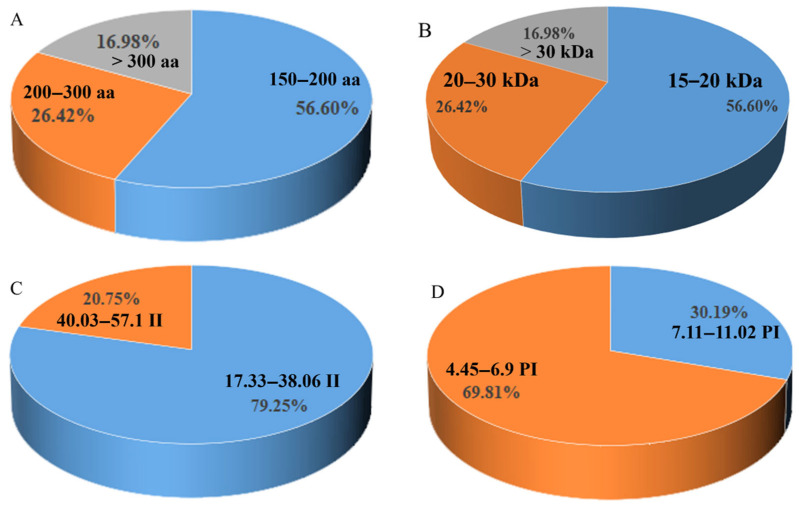
Analysis of physicochemical properties of *S. bicolor* DIR protein. (**A**). Statistics of amino acid length of *S. bicolor* DIR protein. (**B**). Statistics of molecular weights of *S. bicolor* DIR protein. (**C**). Statistics of instability index of *S. bicolor* DIR protein. (**D**). Statistics of isoelectric point of *S. bicolor* DIR protein.

Through HMMER search and domain validation, a total of 53 members of the *DIR* gene family were identified at the *S. bicolor* genome-wide level (Table S1). Based on their chromosomal distribution, these genes were designated as SbDIR1 to SbDIR53. Sequence analysis revealed that the encoded proteins of *S. bicolor* DIR genes varied in length from 157 to 819 amino acids, with molecular weights ranging from 16.75 to 91.92 kDa and isoelectric points (pI) between 4.45 and 11.02 (Figure 1 and Table S1). Furthermore, proteins with an isoelectric point of less than 7 accounted for 69.81% of the total, suggesting that the majority of *S. bicolor* DIR proteins are acidic. Interestingly, we observed that 79.25% of *S. bicolor* DIR proteins exhibited a stability coefficient of less than 40, suggesting that most of these proteins are unstable (Figure 1).

**Figure 2:**
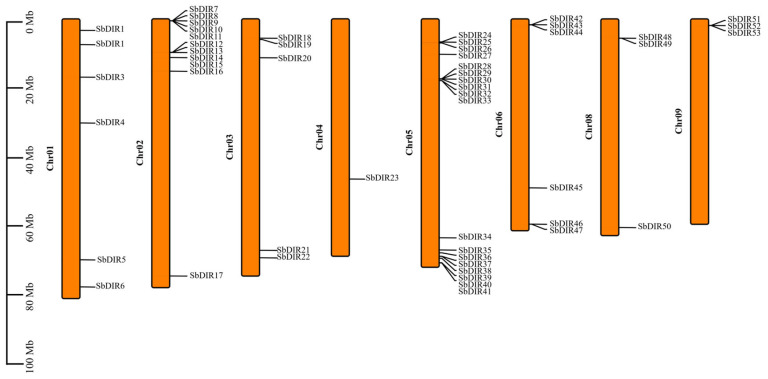
Chromosomal distribution of *DIR* genes in *S. bicolor*.

The analysis of chromosomal localization revealed that the 53 *SbDIR* genes were unevenly distributed across the eight chromosomes (Figure 2). Chromosome 5 exhibited the highest number of *SbDIR* genes, totaling 18, followed by chromosome 2 with 11. Chromosomes 8 and 9 each contained three DIR genes, while chromosomes 1 and 6 each harbored six. Notably, no *SbDIR* genes were detected on chromosome 7.

### 3.2. Structural and Conserved Motif Analysis of S. bicolor DIR Genes

**Figure 3:**
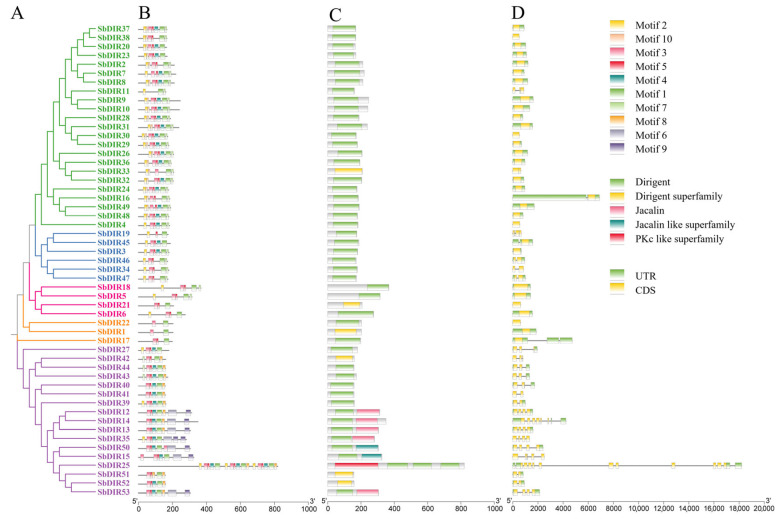
Phylogenetic, conserved motif, domain, and gene structure analysis of sorghum DIR. (**A**) Phylogenetic analysis of sorghum DIR proteins. The neighbor-joining (NJ) tree was constructed using MEGA 7 with 1000 bootstrap replicates. (**B**) Conserved motif analysis of sorghum DIR proteins. Ten motifs were identified using the online tool MEME with default parameters. (**C**) Conserved domain analysis of sorghum DIR proteins. Domains were predicted using the NCBI-CDD database. (**D**) Intron–exon structure of sorghum DIR genes. Visualization was performed using TBtools v2.121.

Gene structure analysis indicated that the number of introns in *S. bicolor* DIR genes varied from 1 to 14 (Figure 2). Notably, SbDIR1, SbDIR2, SbDIR5, SbDIR6, SbDIR7, SbDIR8, SbDIR9, SbDIR10, SbDIR16, SbDIR18, SbDIR20, SbDIR21, SbDIR22, SbDIR23, SbDIR24, SbDIR26, SbDIR28, SbDIR29, SbDIR30, SbDIR31, SbDIR32, SbDIR33, SbDIR36, SbDIR37, SbDIR38, SbDIR45, SbDIR48, and SbDIR49 were found lack introns, representing typical single-exon genes, while others genes contained varying numbers of introns (Figure 3). Conserved motif analysis identified 10 conserved motifs (Motif1–10) (Figure 3). Most DIR proteins contained Motif1, Motif2, Motif3, Motif4, Motif5, and Motif10, which are highly conserved within the DIR domain and may be critical for protein functionality. The composition of motifs exhibited subgroup specificity; for example, Group I members (SbDIR12, SbDIR13, SbDIR14, SbDIR15, SbDIR35 and SbDIR50) uniquely contained Motif6 and Motif9, while Motif8 was present in SbDIR12, SbDIR13, SbDIR14, SbDIR50–SbDIR53, and SbDIR39–SbDIR44 (Figure 3). Domain analysis revealed that certain DIR genes, such as SbDIR12, SbDIR13, SbDIR14, SbDIR35, and SbDIR53, possessed both Dirigent and Jacalin domains, suggesting potential functional complexity (Figure 3).

### 3.3. Phylogenetic Analysis of the S. bicolor DIR Gene Family

**Figure 4:**
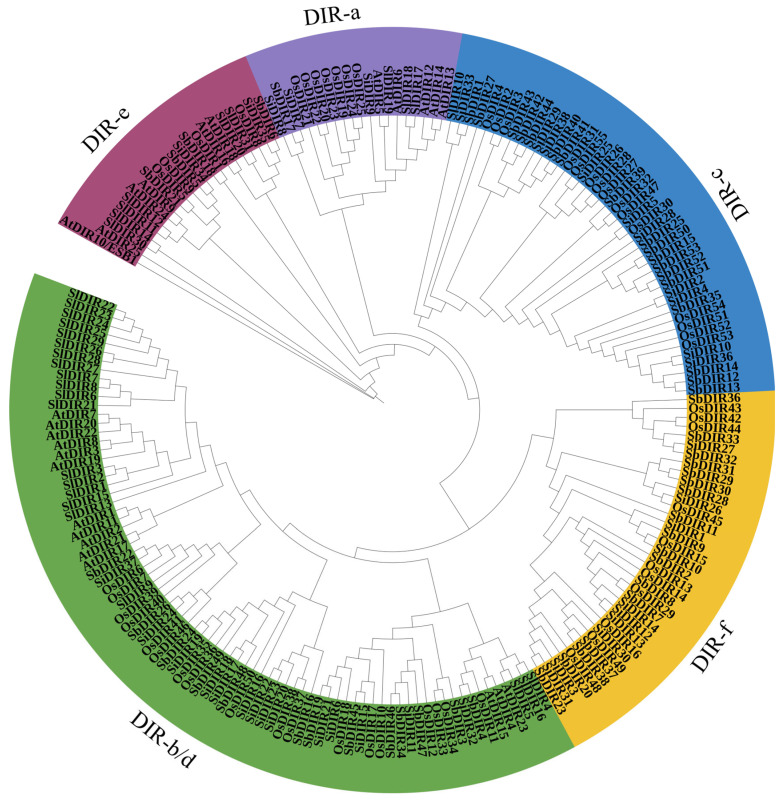
Phylogenetic analysis of DIR proteins in *S. bicolor*, *A. thaliana*, *S. lycopersicum*, *O. sativa*, *S. italica*, and *Z. mays*. The evolutionary tree was constructed using the neighbor-joining (NJ) method with 1000 bootstrap replicates in MEGA 7. DIR proteins were classified into five subfamilies: DIR-a, DIR-b/d, DIR-c, DIR-e, and DIR-f, represented by purple, green, blue, red, and yellow branches, respectively.

To elucidate evolutionary relationships, a phylogenetic tree was constructed using DIR proteins from *S. bicolor*, *A. thaliana*, *S. lycopersicum*, *O. sativa*, *S. italica*, and *Z. mays* (Figure 4). The DIR proteins were classified into five major subfamilies: DIR-a, DIR-b/d, DIR-c, DIR-e, and DIR-f. The DIR proteins of *S. bicolor* were distributed across all subfamilies, with one in DIR-a, thirteen in DIR-b/d, eighteen in DIR-c, four in DIR-e, and seventeen in DIR-f. Notably, the ubiquitous presence of DIR-a, DIR-b/d, and DIR-e across monocot-dicot lineages implies ancient evolutionary origins predating the divergence of angiosperms. Their conservation, potentially maintained by purifying selection, suggests that these subfamilies encode core functions critical for plant survival (Figure 4). In contrast, lineage-specific subfamilies (e.g., DIR-c/f in monocots) may have arisen through gene duplication and neofunctionalization to adapt to ecological niches (Figure 4).

### 3.4. Tandem Duplication Acts as the Primary Driver for the Expansion of the SbDIR Gene Family

**Figure 5:**
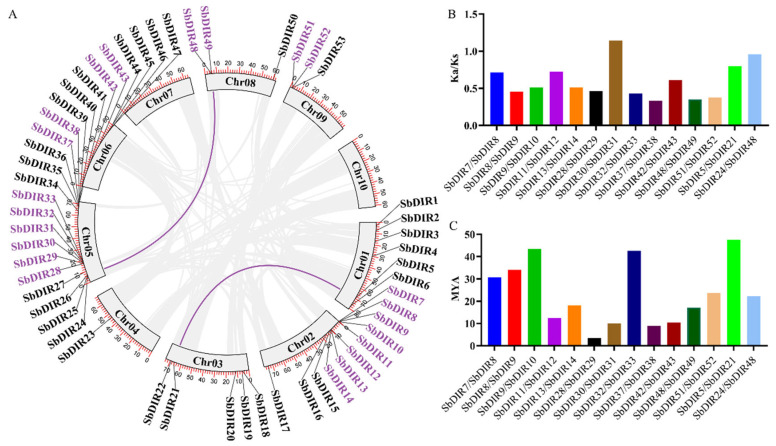
Analysis of gene duplication events, Ka/Ks ratios, and divergence time of *DIR* genes in sorghum. (**A**) Gene duplication events of sorghum DIR genes. Whole-genome duplication (WGD) events are indicated by purple lines, and tandem duplicated genes are labeled with purple gene IDs. (**B**) Ka/Ks analysis of *S. bicolor DIR* genes. The ratio of nonsynonymous (Ka) to synonymous (Ks) substitutions was calculated to assess selection pressure. (**C**) Divergence time estimation of *S. bicolor DIR* genes. The divergence time of duplicated gene pairs was inferred based on molecular clock assumptions.

The expansion of gene families is a key mechanism driving plant adaptive evolution, primarily mediated by tandem duplication (TD) and whole-genome duplication (WGD) [31,32]. In the evolutionary trajectory of the *SbDIR* gene family, TD has played a predominant role (Figure 5). The SbDIR gene family exhibits a distinct clustered genomic distribution, a hallmark of TD events. This process generates highly homologous gene copies at adjacent loci, which may subsequently undergo functional diversification through subfunctionalization (partitioning of ancestral functions) or neofunctionalization (acquisition of novel functions), thereby enhancing plant adaptability to environmental stresses. Compared to WGD, tandem duplication preferentially generates localized gene clusters, which may facilitate rapid responses to specific biotic (e.g., pathogen invasion) or abiotic (e.g., drought) challenges. The DIR gene family is critically involved in plant secondary metabolism (e.g., lignin biosynthesis) and stress adaptation.

The Ka/Ks ratio, a key metric for evaluating evolutionary selection pressure, indicates that the sorghum *DIR* gene family has predominantly undergone purifying selection (Ka/Ks < 1) (Figure 5 and Table S2). This suggests that most nonsynonymous mutations are selectively removed to preserve functional conservation. Such constraints likely reflect the essential biological roles of DIR proteins, including lignin polymerization, defense signaling, and cell wall reinforcement. Although tandem duplication increases gene copy number, functional constraints restrict rapid divergence among new copies, ensuring the evolutionary stability of the gene family.

### 3.5. Synteny Analysis of S. bicolor and Other Species

**Figure 6:**
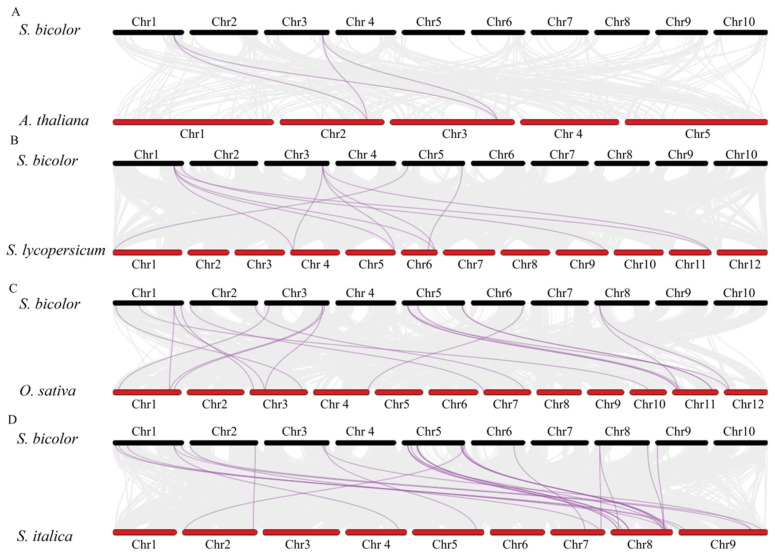
Synteny analysis of sorghum *DIR* genes with other species. (**A**) Synteny between *S. bicolor* and *A. thaliana* *DIR* genes. (**B**) Synteny between *S. bicolor* and *S. lycopersicum* *DIR* genes. (**C**) Synteny between *S. bicolor* and *O. sativa* *DIR* genes. (**D**) Synteny between *S. bicolor* and *S. italica DIR* genes. The purple line represents collinear gene pairs.

To elucidate the evolutionary trajectory of the DIR gene family in sorghum, this study performed whole-genome synteny analysis using MCScanX between *S. bicolor* and representative species, including the dicot model plant *A. thaliana*, monocot crops *O. sativa* and *S. italica*, as well as the solanaceous crop *S. lycopersicum*. A total of 4 (*S. bicolor*–*A. thaliana*), 11 (*S. bicolor*–*S. lycopersicum*), 20 (*S. bicolor*–*O. sativa*), and 27 (*S. bicolor*–*S. italica*) DIR homologous gene pairs were identified (Figure 6). Notably, foxtail millet and sorghum, as closely related species within the Poaceae family, exhibited significantly more syntenic gene pairs than other species (27 vs. 4–20), indicating that the *DIR* gene family retained higher genomic structural conservation after monocot–dicot divergence.

Further analysis revealed “one-to-many” homologous relationships between certain sorghum *DIR* genes and multiple species. For instance, *SbDIR5* showed synteny with both *SlDIR11*, *SlDIR12*, *SlDIR14*, and *SlDIR30* in *S. lycopersicum*. Similarly, *SbDIR48* corresponded to three homologs in *O. sativa* (*OsDIR35* and *OsDIR50*) and two in *S. italica* (*SiDIR16* and *SiDIR19*). Strikingly, within the *S. bicolor*–*A. thaliana* syntenic gene pairs, members of the DIR-e subfamily accounted for 100% of the matches, while no homologs were detected for the DIR-c/f subfamilies. This observation aligns with the cross-lineage conservation of DIR-e and the monocot-specific diversification of DIR-c/f revealed by phylogenetic analysis, suggesting distinct evolutionary selection pressures acting on different DIR subfamilies during the plant.

### 3.6. Cis-Acting Element Analysis in Promoter Regions of S. bicolor DIR Genes

**Figure 7:**
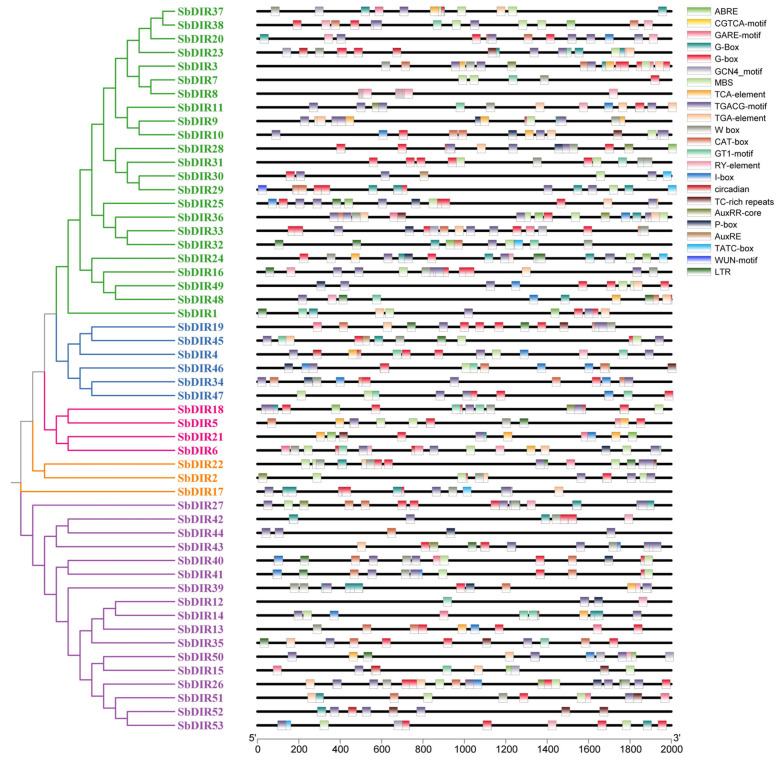
Analysis of cis-regulatory elements in the promoter regions of the *S. bicolor* DIR gene family.

The analysis of cis-acting elements in the promoter regions of *S. bicolor* DIR genes revealed a plethora of motifs associated with hormone response, stress adaptation, and light regulation (Figure 7 and Table S3). The identified hormone-responsive elements included ABRE (abscisic acid), TCA-element (salicylic acid), CGTCA/TGACG-motifs (methyl jasmonate), TGA-element/AuxRR-core (auxin), and P-box/GARE-motif (gibberellin). Stress-responsive elements comprised MBS (drought), LTR (low temperature), and GT1-motif (salt stress). Additionally, light-responsive elements, such as G-box and I-box, were also prevalent. This study demonstrated that the promoter regions of *SbDIR3*, *SbDIR4*, *SbDIR5*, *SbDIR6*, *SbDIR9*, *SbDIR10*, *SbDIR21*, *SbDIR24*, *SbDIR25*, *SbDIR37*, *SbDIR39*, *SbDIR48*, and *SbDIR50* contain both salicylic acid (SA)- and methyl jasmonate (MeJA)-responsive elements, suggesting their potential roles in sorghum disease resistance through SA-MeJA signaling crosstalk. Further analysis revealed combinatorial cis-acting elements in the promoters of certain *DIR* genes, such as the pairing of abscisic acid (ABA) with auxin or SA, implying their ability to integrate diverse hormonal signaling networks. Notably, 92% of *DIR* genes possess ABA-responsive elements, underscoring their critical involvement in ABA-mediated stress adaptation in sorghum. These findings suggest that *S. bicolor DIR* genes may participate in various hormone signaling pathways, stress responses, and photoregulation.

### 3.7. Tissue-Specific Expression and Stress Response Patterns of S. bicolor DIR Genes

**Figure 8:**
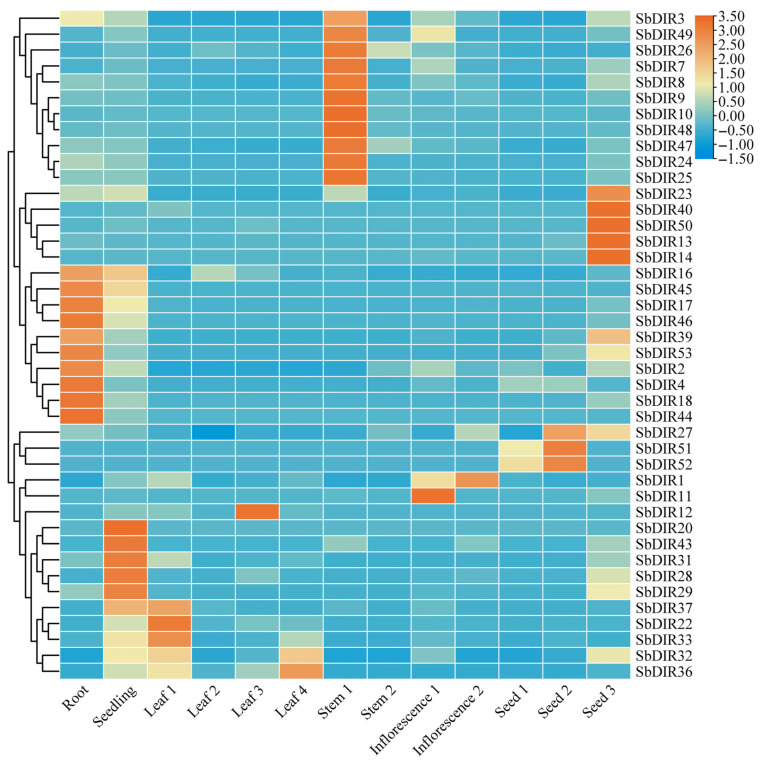
The heat map shows the expression level of the *S. bicolor DIR* gene in different tissues.

Transcriptomic data revealed distinct tissue-specific expression patterns among DIR genes (Figure 8 and Table S4). SbDIR8, SbDIR9, SbDIR13, SbDIR16, SbDIR24, SbDIR26, SbDIR27, SbDIR47, and SbDIR49 were found to be expressed ubiquitously across roots, stems, leaves, flowers, and seeds. Conversely, others genes exhibited tissue-preferential expression: SbDIR16, SbDIR45, SbDIR17, SbDIR46, SbDIR39, SbDIR53, SbDIR2, SbDIR4, SbDIR18, and SbDIR44 showed root-dominant expression; SbDIR13, SbDIR14, SbDIR23, SbDIR40, and SbDIR50 were highly expressed in seeds; while SbDIR3, SbDIR49, SbDIR26, SbDIR7, SbDIR8, SbDIR9, SbDIR10, SbDIR47, SbDIR48, SbDIR24, and SbDIR25 displayed leaf-biased expression. These results suggest that the *SbDIR* gene family plays a crucial role in regulating plant growth and development.

### 3.8. The Expression Level of the SbDIR Gene Under PEG and NaCl Stresses

**Figure 9:**
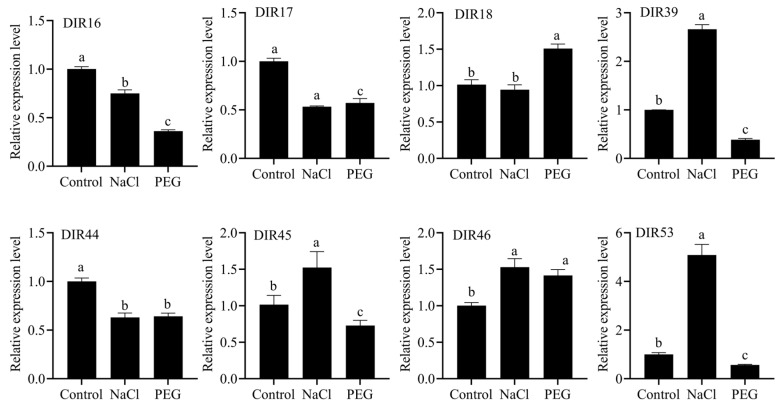
Analysis of the expression levels of *DIR16*, *DIR17*, *DIR18*, *DIR39*, *DIR44*, *DIR45*, *DIR46*, and *DIR53* after treatment with NaCl and PEG. All data are means ± sd (n ≥ 3). Letters a, b and c represent statistical significance, *p* < 0.05.

To explore the expression pattern of the *DIR* gene in sorghum under drought and salt stress, we randomly selected eight *SbDIR* genes that exhibited high expression levels in the roots (Figure 8) to analyze their expression levels via RT-qPCR. The results are shown in Figure 9; the expression levels of several *DIR* genes significantly changed under NaCl and PEG treatment. Under NaCl treatment, the expressions of genes such as *SbDIR39*, *SbDIR45*, *SbDIR46*, and *SbDIR53* were up-regulated, while the expression levels of *SbDIR16*, *SbDIR17*, and *SbDIR44* were down-regulated. In contrast, under PEG treatment, the expressions of genes such as *SbDIR18* and *SbDIR46* were up-regulated, while the expression of *SbDIR16*, *SbDIR17*, *SbDIR39*, *SbDIR45,* and *SbDIR53* were down-regulated. Notably, the *SbDIR16*, *SbDIR17*, and *SbDIR44* genes exhibited down-regulation following both drought and salt stress treatment. These findings suggest that the DIR gene in sorghum may play a role in the plant’s response to salt and drought stress.

### 3.9. Gene Co-Expression Analysis

**Figure 10:**
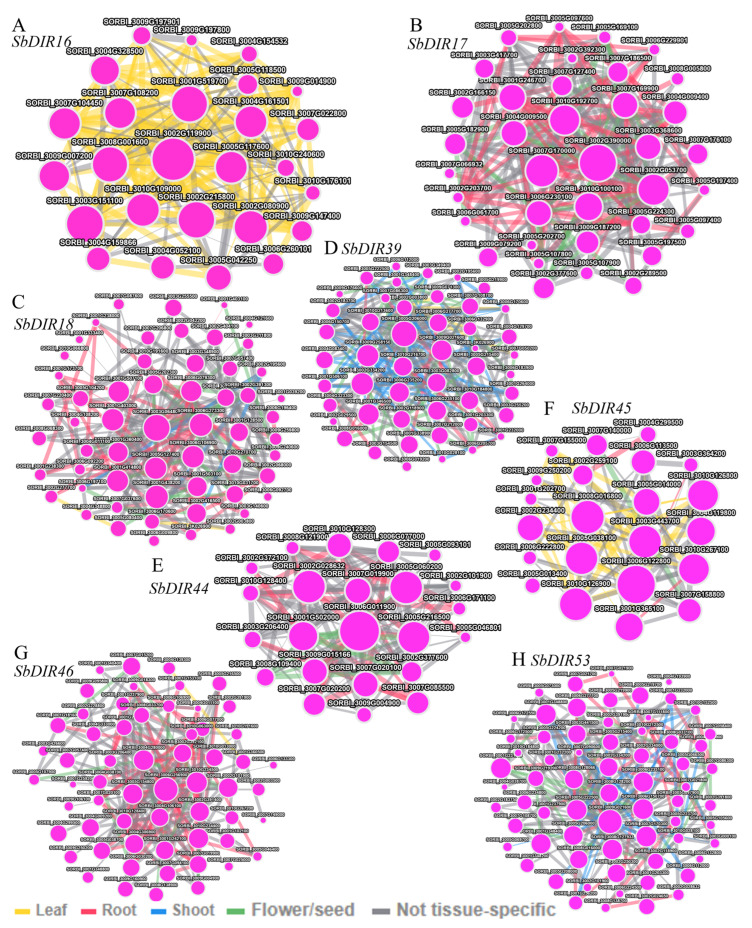
Co-expression network of *SbDIR16* (**A**), *SbDIR17* (**B**), *SbDIR18* (**C**), *SbDIR39* (**D**), *SbDIR44* (**E**), *SbDIR45* (**F**), *SbDIR46* (**G**), and *SbDIR53* (**H**). Dots represent genes, and lines indicate that they have co-expression relationship.

Co-expression analysis helps identify genes that are closely co-regulated during physiological processes. In this study, we used the PlantNexus database [33] to create a co-expression network focused on the following genes: *SbDIR16*, *SbDIR17*, *SbDIR18*, *SbDIR39*, *SbDIR44*, *SbDIR45*, *SbDIR46*, and *SbDIR53*. As shown in Figure 10, eight co-expression networks were obtained. The network centered on *SbDIR53* is the largest, comprising 66 genes. Next is the network centered on *SbDIR46*, which includes 58 genes. In contrast, the networks centered on *SbDIR44* and *SbDIR45* are the smallest, each containing 22 genes. Figure 10 shows that the network centered on *SbDIR17*, *SbDIR18*, *SbDIR44*, *SbDIR46*, and *SbDIR53* is significantly enriched in root expression. This suggests that these genes may play a role in root development. The root system primarily absorbs water and nutrients in plants and serves as the first line of defense against soil stressors, such as salinity and drought. The deposition of lignin in root cell walls enhances their mechanical strength, reduces water loss, and prevents invasion by soil pathogens. Therefore, these root-specific *SbDIR* genes likely regulate the synthesis and deposition of lignin in sorghum roots. Overall, these findings reveal an intriguing phenomenon that warrants further investigation.

## 4. Discussion

### 4.1. Identification and Characterization of the DIR Gene Family in Sorghum

This study systematically identified 53 *DIR* gene family members in the sorghum genome, revealing a notable species-specific distribution pattern. Compared with tetraploid *G. hirsutum* (105) [34] and *O. sativa*, (55) [10], the sorghum DIR family exhibits a relatively smaller size, yet significantly larger than those in *A. thaliana* and *Capsicum annuum* (*C. annuum*) [7,35]. These interspecific variations may arise from differential genome polyploidization events (e.g., A/D subgenome doubling in cotton) and ecological adaptation-driven functional selection pressures (e.g., specialized lignin biosynthesis requirements in monocot Poaceae species). Integrated phylogenetic and gene structure analyses demonstrated distinct exon–intron architectures among SbDIR subfamilies: DIR-a subfamily predominantly retained single-exon structures, whereas DIR-c subfamily displayed complex exon-intron configurations. These complex exon–intron configuration genes might be adapted to adverse environments (e.g., the validated 5-fold up-regulation of SbDIR53 under salt stress), an evolutionary advantage aligning with sorghum’s adaptive strategy as a C4 pioneer crop in arid habitats. Furthermore, the intronless architecture may maintain stable functional gene expression through resistance to epigenetic silencing mechanisms (e.g., avoiding siRNA-mediated intronic region methylation). However, the precise molecular mechanisms warrant further investigation.

### 4.2. Phylogenetic Analysis of the SbDIR Gene Family

Phylogenetic analysis serves as a pivotal method for studying the origin and evolution of gene families and plays a critical role in elucidating the origin and diversification mechanisms of plant functional proteins [36]. In this study, by constructing a phylogenetic tree of DIR proteins from sorghum, Arabidopsis, tomato, rice, and foxtail millet, we classified DIR proteins into five subfamilies. Among these, the DIR-c and DIR-f subfamilies are uniquely present in monocots, while the DIR-e, DIR-a, and DIR-b/d subfamilies are conserved in both monocots and dicots. This finding suggests that DIR genes existed before the divergence of plant species and underwent gene duplication and functional differentiation during evolution. SbDIR5, SbDIR21, SbDIR6, and SbDIR18 are clustered into the DIR-e subfamily along with AtDIR16, AtDIR18, AtDIR9, AtDIR24, AtDIR25, and AtESB1 from Arabidopsis. In Arabidopsis, the DIR-e subfamily is involved in Casparian strip formation, suggesting that SbDIR5, SbDIR21, SbDIR6, and SbDIR18 may play critical roles in the development of the Casparian strip in sorghum. This functional conservation highlights their potential importance in regulating root endodermal barrier formation and nutrient transport in sorghum [37]. Gene duplication is a key mechanism for generating novel genes and expanding gene family membership [10]. Evolutionary dynamics analysis revealed that SbDIR genes primarily expanded through tandem duplication events, a pattern consistent with that observed in rice. The distribution of SbDIR family members across subfamilies likely reflects evolutionary gene duplication events and functional divergence pathways. Additionally, variations in conserved motifs and gene structures among DIR protein subfamilies provide critical insights into the evolution and functional specialization of the DIR gene family.

### 4.3. Analysis of Cis-Acting Elements in the Promoter Regions of SbDIR Genes

Cis-acting elements are critical regulatory components of gene expression, modulating transcriptional processes through interactions with transcription factors. Analysis of the promoter regions of *SbDIR* genes revealed an abundance of hormone-responsive elements, stress-responsive elements, and light-responsive elements. The presence of these cis-acting elements suggests that *SbDIR* genes may participate in diverse hormone signaling pathways, stress response mechanisms, and light-regulated processes. For example, abscisic acid (ABA), a key phytohormone in plant stress adaptation, is associated with ABRE (ABA-responsive element), which is widely distributed in promoters of stress-responsive genes. In this study, 90.57% of sorghum *DIR* gene promoters harbored ABRE elements, indicating their potential involvement in ABA-mediated stress response pathways, which is consistent with the previous reports in *S. italica* [38]. Additionally, the identification of hormone-responsive elements for methyl jasmonate (MeJA), auxin, and gibberellin (GA) implies that *DIR* genes may function in the cross-regulation of multiple hormone signaling cascades. These findings highlight the regulatory complexity and adaptive versatility of the *DIR* gene family in sorghum.

### 4.4. Tissue-Specific Expression Patterns and Stress Response Analysis of SbDIR Genes

Lignin, a fundamental component of plant cell walls, plays an indispensable role in plant growth and development, structural support, water transport, and stress resistance [39]. It not only provides mechanical strength to plant cell walls, enabling them to withstand external mechanical stress and pathogen invasion, but also regulates cell wall permeability, thereby influencing the efficiency of water and nutrient transport [39]. Furthermore, during plant responses to abiotic stresses such as drought and salinity, lignin synthesis and deposition enhance cellular stress resistance barriers, serving as a crucial physiological foundation for plant adaptation to varying environmental conditions [16]. DIR proteins are key regulatory factors in lignin biosynthesis in plants [3,16]. They influence the structure, composition, and biological function of lignin with their specificity and precision, playing an irreplaceable role in plant growth, development, and environmental adaptation [40]. Transcriptomic data analysis in this study revealed tissue-specific expression patterns among *SbDIR* genes, consistent with previous findings in rice, Arabidopsis, and *S. italica* [7,38]. While some SbDIR genes were broadly expressed across tissues such as roots, stems, leaves, flowers, and seeds, others exhibited high tissue-specific expression, suggesting functional diversification during sorghum growth and development. For example, *SbDIR16*, *SbDIR45*, *SbDIR17*, *SbDIR46*, *SbDIR39*, *SbDIR53*, *SbDIR2*, *SbDIR4*, *SbDIR18*, and *SbDIR44* showed significantly higher expression in roots compared to other tissues, potentially implicating their roles in root development. The root system is the organ primarily responsible for absorbing water and nutrients in plants [41]. It also serves as the first line of defense against soil stressors, such as salinity and drought [42]. The deposition of lignin in root cell walls increases their mechanical strength, decreases water loss, and prevents invasion by soil pathogens [43]. Therefore, these root-specific *SbDIR* genes likely regulate the synthesis and deposition of lignin in sorghum roots. This has been confirmed in corn, where the *ZmDIR5* mutation also inhibits phenylalanine biosynthesis [17].

Previous studies have shown that *DIR* genes are expressed differently in response to various abiotic stresses [7,10,35]. We found that *SbDIR16/17/18/39/44/45/46/53* genes displayed differential expression under salt and drought stress, indicating their potential roles in plant stress responses. On the other hand, we found cis-regulatory elements related to responses to adverse conditions, such as drought, in the promoter regions of these genes. This finding further indicates that these genes may play a role in responses to adverse conditions, such as drought. Wu et al. (2009) reported a similar finding in their study [44]. Additionally, increased expression of *ScDIR* genes was observed in sugarcane in response to NaCl, PEG, and oxidative stress treatments [45]. These findings underscore the potential role of *SbDIRs* in the regulation of abiotic stress.

Lignin, a critical component of plant cell walls, plays a pivotal role in stress adaptation [39]. *SbDIR* genes may enhance stress tolerance by regulating lignin biosynthesis and deposition, thereby modulating cell wall structure and function. For instance, *SbDIR39*, *SbDIR45*, *SbDIR46*, and *SbDIR53* were up-regulated under salt stress, potentially promoting lignin synthesis and deposition to reinforce cell wall integrity and improve salt tolerance. However, the precise molecular mechanisms remain to be elucidated. This hypothesis is supported by studies in other species: overexpression of *GmDIR22* in *G. max* enhances resistance to P. sojae by boosting lignan biosynthesis [18]. Overexpression of *GhDIR1* in cotton improves resistance to V. dahliae via increased lignan production [46]. *OsDIR55* in *O. sativa* enhances salt tolerance by regulating root lignification [5,16]. A recent study published in *Science* confirms our hypothesis that *AtDPs* mutants exhibit significant abnormalities in lignin deposition at the Casparian strip [37]. This is accompanied by a loss of tight junctions between the cell membrane and the cell wall at this site. These abnormalities compromise the function of the Casparian strip as a barrier to water and mineral element diffusion, severely disrupting mineral element homeostasis within the plant. Consequently, this reduction in homeostasis diminishes the plant’s ability to adapt to various abiotic stress conditions, including salt stress, osmotic stress, and low humidity stress.

While this study provides bioinformatic predictions, functional validation of key genes (e.g., via transgenic overexpression or CRISPR-Cas9 knockout) is essential to confirm their roles in stress adaptation and lignin-mediated pathways in the future. This study found that the expression levels of *SbDIR39* and *SbDIR53* were upregulated by 2.8-fold and 5-fold, respectively, under 150 mM NaCl stress, directly demonstrating their positive role in the salt stress response. Together with promoter cis-element analysis, which contains numerous hormone and stress response elements, these two genes can serve as potential targets for gene editing. For instance, enhancing their expression using CRISPR technology could improve sorghum’s survival rate in saline-alkali soils. Thus, these genes could be introduced into major cultivated varieties through hybrid breeding to create new salt-tolerant varieties.

## 5. Conclusions

We systematically identified and characterized 53 *DIR* genes in *S. bicolor*. Phylogenetic analysis classified them into five subfamilies, with members of the same subclade sharing conserved exon–intron structures and motifs. Collinearity analysis indicated tandem duplication as the dominant driver of *SbDIR* family expansion. RNA-seq revealed 10 root-specific *SbDIR* genes (*SbDIR2/4/16/17/18/39/44/45/46/53*), which likely regulate root lignin synthesis and deposition to optimize root morphology and stress resistance, highlighting lignin’s role in underground organ development and environmental adaptation. RT-qPCR showed stress-responsive divergence: *SbDIR16/17/44* were downregulated under salt and PEG stress, while *SbDIR39/53* were upregulated under PEG stress. These genes may mediate abiotic stress responses by altering lignin synthesis or composition via regulating monomer coupling. Our findings enhance understanding of *SbDIR* evolution and diversification, providing valuable genetic resources for improving *S. bicolor* root traits and stress tolerance through molecular breeding.

## Data Availability

Rest assured, I have ensured that all data, materials, software applications, and custom code supporting the claims made in this article are in full compliance with field standards. Data are contained within the article or Supplementary Materials. The datasets about the Public RNA-seq data during the current study are available in the *S. bicolor* Genome and Mutant Bank SGMD database (https://sorghum.genetics.ac.cn/SGMD/, accessed on 25 April 2025).

## Supplementary Materials

The following supporting information can be downloaded at https://www.mdpi.com/article/10.3390/genes16080973/s1, Table S1: Physical and chemical properties analysis of SbDIR protein; Table S2: Ka/Ks values of duplicate gene pairs in S. bicolor; Table S3: Distribution of cis-acting elements in the SbDIR gene promoter; Table S4: Analysis of tissue-specific expression patterns; Table S5: RT-qPCR primers for the SbDIR family.
